# A novel capsule-based self-recovery system with a chloride ion trigger

**DOI:** 10.1038/srep10866

**Published:** 2015-06-08

**Authors:** Wei Xiong, Jiaoning Tang, Guangming Zhu, Ningxu Han, Erik Schlangen, Biqin Dong, Xianfeng Wang, Feng Xing

**Affiliations:** 1 Department of Civil Engineering, Guangdong Provincial Key Laboratory of Durability for Marine Civil Engineering, Shenzhen University, Shenzhen 518060, PR China; 2 Shenzhen Key Laboratory of Special Functional Materials, College of Materials Science and Engineering, Shenzhen University, Shenzhen 518060, PR China; 3 Delft University of Technology, Faculty of Civil Engineering and Geosciences, Micromechanics Laboratory (MICROLAB), Stevinweg 1, 2628 CN Delft, The Netherlands

## Abstract

Steel is prone to corrosion induced by chloride ions, which is a serious threat to reinforced concrete structures, especially in marine environments. In this work, we report a novel capsule-based self-recovery system that utilizes chloride ions as a trigger. These capsules, which are functionalized via a smart response to chloride ions, are fabricated using a silver alginate hydrogel that disintegrates upon contact with chloride ions, and thereby releases the activated core materials. The experimental results show that the smart capsules respond to a very low concentration of chloride ions (0.1 wt%). Therefore, we believe that this novel capsule-based self-recovery system will exhibit a promising prospect for self-healing or corrosion inhibition applications.

Microencapsulation is an increasingly important technique that has been applied in many fields, such as self-healing materials^1^,^2^,^3^, drug delivery^4^,^5^,^6^, food preservation^7^,^8^,^9^, phase change materials^10^,^11^ and fragrance release^12^,^13^. It is believed that encapsulation technology is a burgeoning research area for self-healing materials, and such technology can heal some cracks which are difficult to repair due to inaccessibility, as well as the cost is very low. However, a significant drawback in current capsule-based self-healing technology is that it is difficult to ensure all capsules will be broken by cracks (Fig. S1a), this depends on the interface bonding strength between the capsule and the matrix, so the healing efficiency will be low.

In anti-corrosion field, as we know, steel bars in reinforcement concrete are prone to corrosion induced by chloride ions, especially the reinforced concrete structure in marine environments^14^. The most commonly adopted strategy to solve this problem is to mix a corrosion inhibitor into concrete. Unfortunately, during the fabrication process of cement slurry, much of the corrosion inhibitor is lost, because of its dissolution and diffusion. It is a feasible method to embed capsules encapsulated corrosion inhibitor substance into matrix to solve the problem. When cracks appear, the capsules will be broken under stress to release the core materials. However, it is also not guaranteed that the capsules will function properly under a mechanical stress trigger. Furthermore, chloride ions can spread through many nano-cracks and channels that exist in the concrete matrix without the formation of micro-cracks (Fig. S1b); thus, when the rebars have been corroded, the capsules may still not start to work. In view of this, specifically, chloride ions will be a desirable trigger in a chloride-attack environment. Therefore, it is necessary to fabricate a kind of smart capsule which can be responsive to chloride ions, this concept can overcome these drawbacks. Many papers have reported a range of stimuli responsive capsules that disintegrate when exposed to physical^15^,^16^,^17^, biological^18^, chemical^19^,^20^ and combination triggers^21^. However, to the best of our knowledge, chloride ions stimuli responsive self-recovery system has not been reported yet. In the present work, we also demonstrate its potential application in cementitious materials.

Chloride ions provide a stable trigger for responsive capsules. A facile method is to fabricate responsive materials containing metal ions (such as Ag^+^, Pb^2+^) which can be precipitated with chloride ions to participate the wall formation of capsules. When these smart responsive capsules contact with chloride ions, the metal ions will be extracted out to disintegrate the capsules (Fig. 1a).

Alginate is a natural anionic polymer acquired from brown algae and has been extensively utilized for biomedical applications^22^. Sodium alginate can be crosslinked with many metal ions (such as Ca^2+^) to form hydrogel. In this experiment, Ag^+^ is chosen to be coordinated with alginate to form the wall materials of capsules, silver alginate (Ag-alg) can also form a characteristic “egg-box” structure^23^. Each alginate molecular chain can be linked with other chains, causing the formation of a three-dimension gel network (Fig. 1b)^24^, which has enough strength to form the wall.

## Results

A range of methods have been utilized to fabricate alginate capsules including microfluidics^25^,^26^,^27^, internal gelation^28^,^29^ and orifice solid bath. In this work, Ag-alginate capsules are fabricated using the orifice solid bath method (Fig. S2)^30^. This approach is a facile synthesis and low cost technique. Its sole requirements are to mix core materials with sodium alginate solution, in addition to encapsulating aqueous substances, if the core materials are oil, surfactant is needed. After stirring, a syringe is used to extract the emulsion, and then squeeze the emulsion out drop by drop, the droplets form capsules gelled by AgNO_3_ solution, the Ag-alginate capsules with oil cores are obtained.

**Figure 2:**
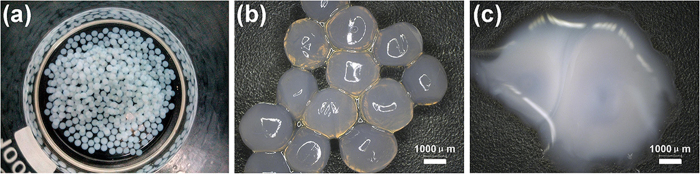
a) Ag-alg capsules b) Optical image of Ag-alg capsules c) The Ag-alginate capsule that disintegrated when exposed to chloride ions.

Figure 2a shows the images of the Ag-alginate capsules. Figure 2b illustrates the magnified microscopic image of Ag-alginate capsules. The average size of capsules is around 2.5 mm. When the Ag-alginate capsules were added with several drops of NaCl 3.5 wt% (the concentration of NaCl in seawater) aqueous solution, the capsules were collapsed entirely in around 1 minute (Fig. 2c). The chloride ions triggering process was recorded in a movie (Supplementary movie 1).

Figure S3 shows the FTIR spectra of Na-alg and Ag-alg compounds. In the spectrum of Na-alg, the stretching vibrations peak of O-H is at 3268 cm^−1^ 31 and the adjacent weak peak 2918 cm^−1^ should be assigned to C–H stretching vibrations of the methyne groups. The C-O-C stretching vibrations absorption brand of saccharide structure is at 1024 cm^−1^ 32. The absorption bands for the symmetric and asymmetric vibrations of carboxylate anions are 1399 cm^−1^ and 1568 cm^−1^, respectively^33^. Comparing the two spectra, it is clear to find the absorption peak at 1568 cm^−1^ shifted to 1598 cm^−1^ in Ag-alg, and the absorption intensity of the peak at 1411 cm^−1^ becomes stronger than the peak at 1399 cm^−1^. These results indicate the binding of Ag with funtional groups contained O^34^. Figure S4 shows the size distributions of Ag-alg beads and Ag-alg capsules. The capsules are uniform, the size distributions both are narrow, and the average diameters are 2.4 mm and 2.5 mm, respectively.

FESEM (field environment scanning electron microscope) was employed to display the morphological features of the Ag-alginate capsule after being freeze dried (Fig. S5a). The porous structure of the surface is observable (Fig. S5b) for the evaporation of water in hydrogel. Figure S5c shows the image of a capsule that was exposed to chloride ions. It is clear that the responsive capsule was collapsed entirely, proving the Ag-alginate capsules can be triggered by chloride ions. Figure S5d shows the surface of the capsule after being triggered, compared to Figure S5b, the porous structure has disappeared. The elements analysis was carried out using energy dispersive X-ray spectroscopy (EDS) measurement, which identified the presence of the C, O, Na and Ag elements. the EDS elemental mapping indicated these elements were uniformly distributed on the surface of Ag-alg capsule (Fig. S6). After the triggering process, the EDS elemental mapping and element analysis were showed in Figure S7. It is noteworthy that the distribution of Ag became sparse for the disintegration of the Ag-alg capsule.

An alternative analysis is necessary for obtaining the chemical valence information of Ag. The XPS (X-ray photoelectron spectroscopy) survey spectrum is shown in Figure S8a. The peaks of Ag 3d_5/2_ and Ag 3d_3/2_ are identified at 368.4 eV and 374.5 eV, respectively. The peaks of Ag 3d_5/2_ and Ag 3d_3/2_ can be further divided into peaks at 368.1, 369.0 eV and 374.0, 374.9 eV, respectively. The peaks at 368.1 and 374.0 eV could be attributed to Ag^+^ 35,36 from Ag_2_O or Ag-alg, whereas those at 369.0 and 374.9 eV could be ascribed to Ag(0)^32^ (Figure S8b).

**Figure 3:**
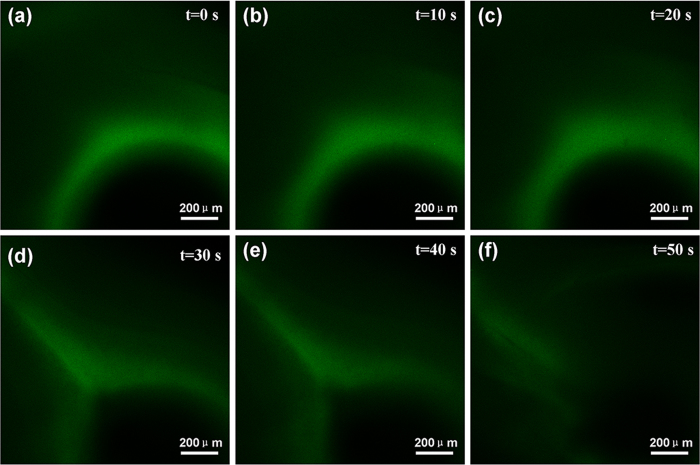
Time-lapse CLSM images showing capsules exposed to chloride ions.

We employ confocal laser scanning microscopy (CLSM) to demonstrate the chloride ions responsive process (Fig. 3a–f). The Ag-alg capsule was labeled with green fluorescent dye - FITC (fluorescein isothiocyanate). The capsule was added with some drops of 3.5 wt% NaCl solution, and then the capsule collapsed. This gradual responsive process proceeds over 50 s. It is in accordance with the preceding responsive experiment. Figure S9 shows the triggering time of Ag-alginate capsules added with different concentration of NaCl solution. The concentration is 0.1 wt%, 1 wt%, 2 wt%, 3 wt%, 4 wt% respectively. Accordingly, the triggering time is 142, 96, 83, 71 and 62 seconds. This proves the Ag-alginate capsules can be triggered at low concentration of chloride ions, the NaCl concentration of seawater is 3.5 wt%, consequently, the capsules can be triggered in marine environments.

**Figure 4:**
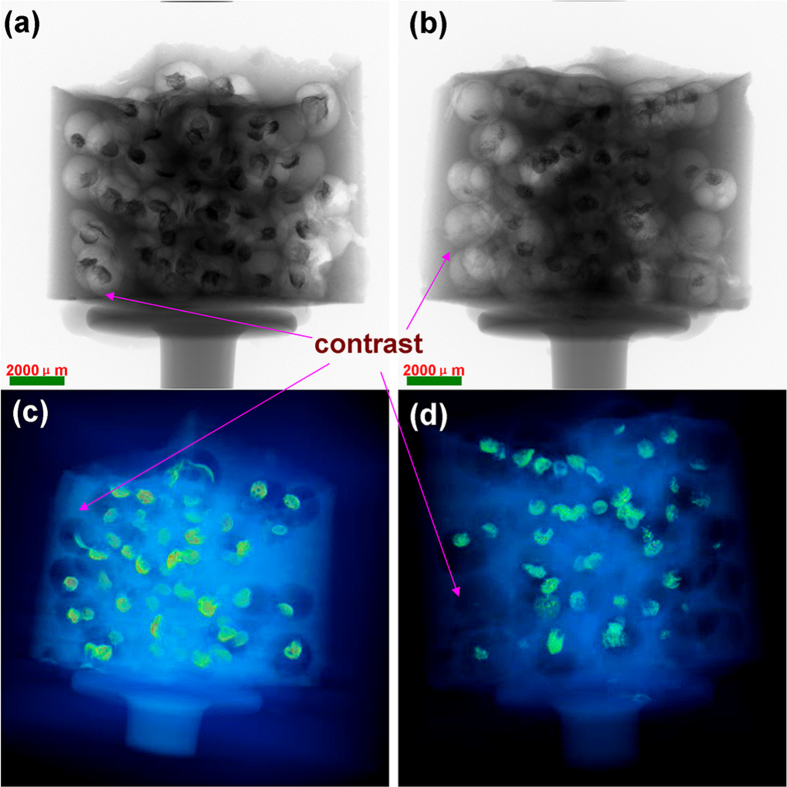
X-ray CT images of concrete a) Concrete specimen and X-ray CT test specimen in the inset, b) Concrete specimen soaked into NaCl solution, c) 3D image of concrete specimen, 4) 3D image of concrete specimen soaked into NaCl solution.

X-ray computed tomography (X-ray CT) is a promising non-destructive technique to demonstrate the inner morphology within non-optically transparent materials. Moreover this versatile characterization approach can offer reconstructing modeling of spatial characteristics and volume fraction in 3D^37^. X-ray CT was employed to detect concrete specimen embedded with chloride ions responsive Ag-alg capsules (Fig. S10). For much more visualized contrast, Ag-alg capsules were not freeze dried. The Ag-alginate capsules became smaller during the concrete specimen hydrated process for the loss of water molecules in the framework of Ag-alginate hydrogel. Figure 4a shows the concrete specimen embedded with Ag-alginate capsules, but not be soaked with NaCl solution, it is observable to identify the shrinkage hydrogel. Figure 4b depicts the concrete specimen exposed to chloride ions environment. It is clear that the capsules near the surface of concrete specimen have disappeared, leaving hollow pores. In X-ray CT 3D model, heavy metals like Ag have a strong absorbency for X-ray, and appear as bright objects in 3D images. Compared with X-ray CT 3D images (Fig. 4c,d), the capsules near the surface of concrete specimen also have disappeared, the contrast is much more observable. (Supplementary movie 2 and movie 3)

## Discussion

In this work, we have introduced a new concept: chloride ions responsive capsule-based self-recovery system. Compared to traditional mechanical stress responsive system, the advantageous features of it are as follows. First, it is difficult to insure the capsules can be broken under mechanical stress (Fig. S1a). On the contrary, chemical responsive capsules can overcome this drawback, when contact with chloride ions, the all capsules will be broken. Second, chloride ions can penetrate into concrete matrix through nano cracks and channels without micro cracks formation (Fig. S1b), the smart capsules can still be responsive to chloride ions without relying on cracks, this has been proved by X-ray CT test.

In summary, we have successfully fabricated smart chloride ions responsive capsules, first introduce them into cementitious materials, and prove the capsules still can be responsive to chloride ions in concrete matrix. This new self-recovery system will reduce deleterious effect of chloride-attack and possesses high broken efficiency and sensitivity advantages. Therefore, we anticipate that this novel self-recovery system will be a promising candidate for building materials and prolong the life of construction and building materials, especially in marine environment.

## Methods

### Ag-alginate capsules fabrication method

1.5 wt% sodium alginate solution emulsified with methyl methacrylate (MMA) and sodium dodecyl benzene sulfonate (SDBS), MMA is the oil core, SDBS is surfactant, we use a syringe to squeeze the emulsion out drop by drop into solid bath - 1 mol·L^−1^ AgNO_3_. After 10 h, the solution was filtered to obtain the capsules, washed with deionized water for several times.

### Chloride ions responsive concentration test

To pick up four Ag-alginate capsules on a piece of smooth glass, added with different concentration of NaCl solution, that is 1 wt%, 2 wt%, 3 wt%, 4 wt%, respectively, the solution volume all are 0.3 mL, dropped by syringe.

### Confocal laser scanning Microscopy

CLSM experiments were adopted to show the process of capsule triggered by chloride ions. Chitosan is labeled by FITC under weak base circumstance (pH value is 9), and then to mix the labeled chitosan into sodium alginate solution, after the fabrication of capsule procedures, we obtain chloride ions responsive capsules labeled with FITC.

### Concrete specimen embedded Ag-alginate capsules

10 g cement was added to 4 g water, the ratio of water to cement is 0.4. The slurry was added to 1 g (10 wt% of cement) Ag-alginate capsules. After 7days, one concrete specimen was tested by X-ray CT, the other concrete specimen was soaked into 20 wt% NaCl (high concentration of NaCl is adopted for saving experiment time) solution for 15days, and then the soaked specimen was tested by X-ray CT.

## Additional Information

**How to cite this article**: Xiong, W. *et al.* A novel capsule-based self-recovery system with a chloride ion trigger. *Sci. Rep.* **5**, 10866; doi: 10.1038/srep10866 (2015).

## Supplementary Material

Supplementary Information

Supplementary Movie 1

Supplementary Movie 2

Supplementary Movie 3

## Figures and Tables

**Figure 1:**
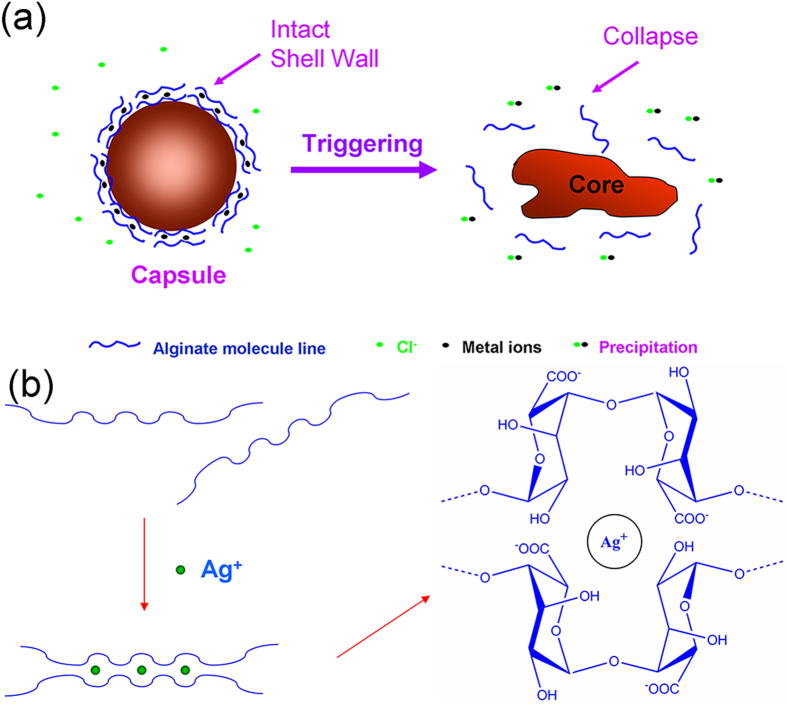
a) Schematic of capsules triggered by chloride ions b) The structure of alginate chelated with Ag^+^
