# Supplementary material for: A novel capsule-based self-recovery system with a chloride ion trigger
Source: Sci Rep. 2015 Jun 8;5:10866. doi: 10.1038/srep10866 (PMC4458884; doi:10.1038/srep10866)
Supplement: Supplementary Information [file srep10866-s1.pdf]

Supplementary Information

A novel capsule-based self-recovery system with chloride ions trigger

Wei Xiong[1,2,3], Jiaoning Tang[2], Guangming Zhu[2], Ningxu Han[1], Erik Schlangen[3], Biqin Dong[1], Xianfeng Wang[1] & Feng Xing[1,*]

[1]Department of Civil Engineering, Guangdong Provincial Key Laboratory of Durability for Marine Civil Engineering, Shenzhen University, Shenzhen 518060, PR China, [2]Shenzhen Key Laboratory of Special Functional Materials, College of Materials Science and Engineering, Shenzhen University, Shenzhen 518060, PR China, [3]Delft University of Technology, Faculty of Civil Engineering and Geosciences, Micromechanics Laboratory (MICROLAB), Stevinweg 1, 2628 CN Delft, The Netherlands.

**Email address:* xingf@szu.edu.cn

Movies

Movie S1: Chloride ions triggering process.

Movie S2: X-ray CT 3D modeling of original concrete matrix embedded with Ag-alg capsules.

Movie S3: X-ray CT 3D modeling of the concrete matrix soaked into NaCl solution.

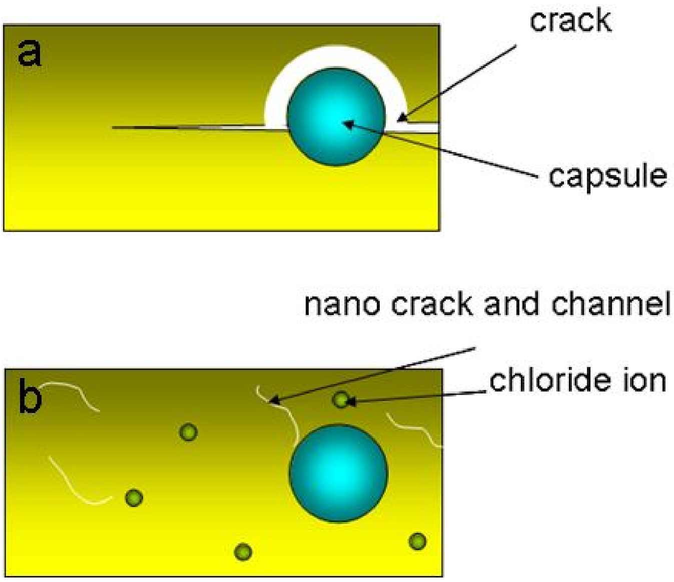

Figure S1. The disadvantages of current healing technology

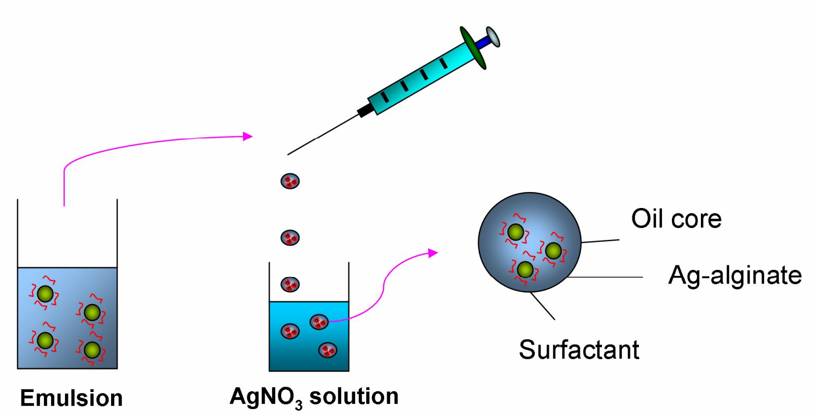

Figure S2. Schematic of Ag-alginate capsules encapsulated oil cores.

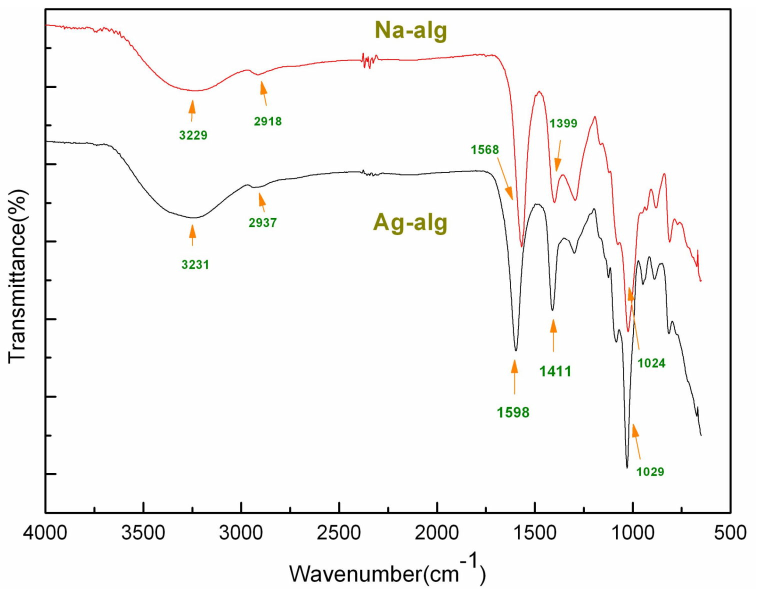

Figure S3. FTIR spectra of Na-alg and Ag-alg.

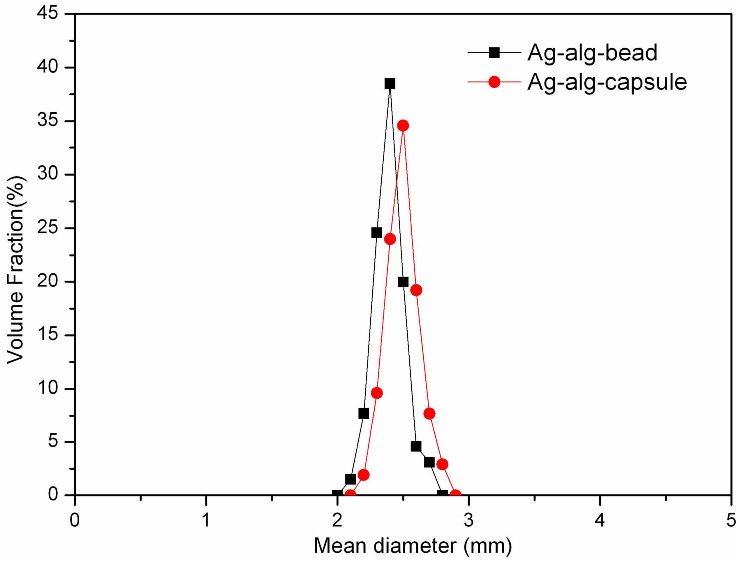

Figure S4. The size distribution of the Ag-alg beads and Ag-alg capsules.

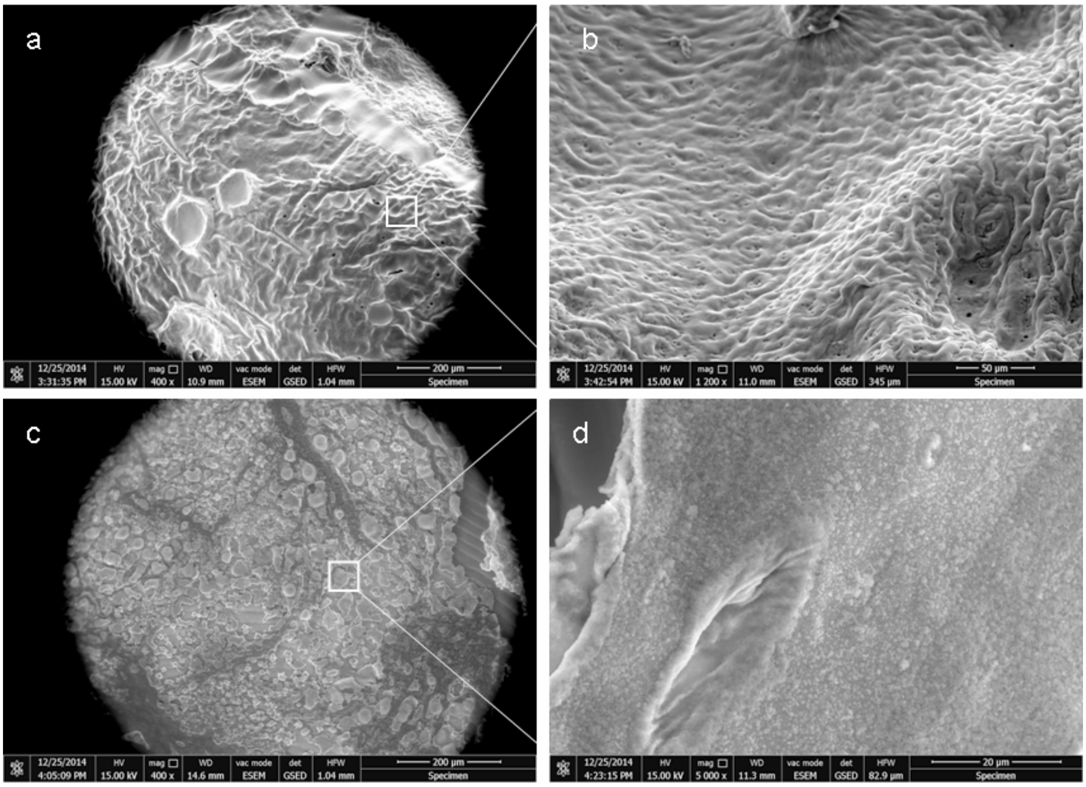

Figure S5. FESEM images of a) Ag-alginate capsule b) the surface of the capsule c) the capsule added NaCl aqueous solution d) the surface after triggering process

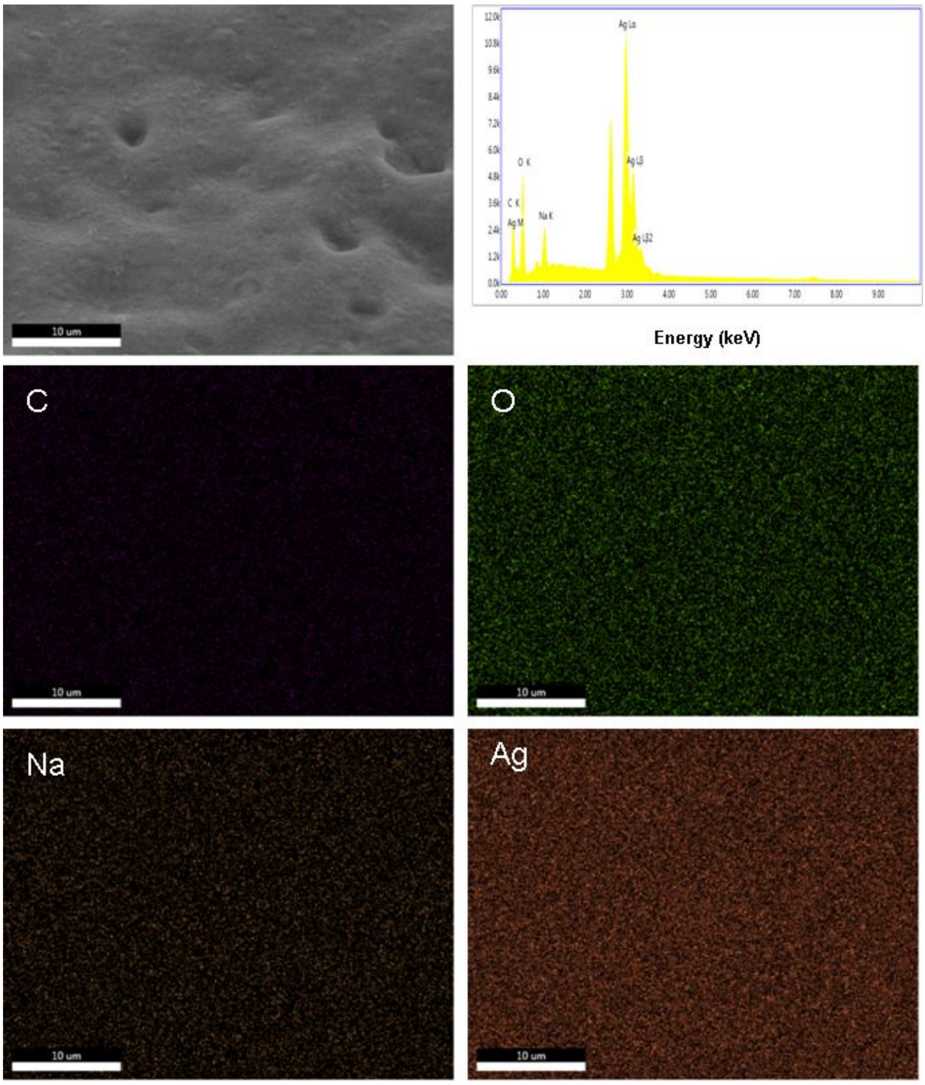

Figure S6. FESEM-EDS compositional elements analysis and mapping images of the Ag-alg capsule

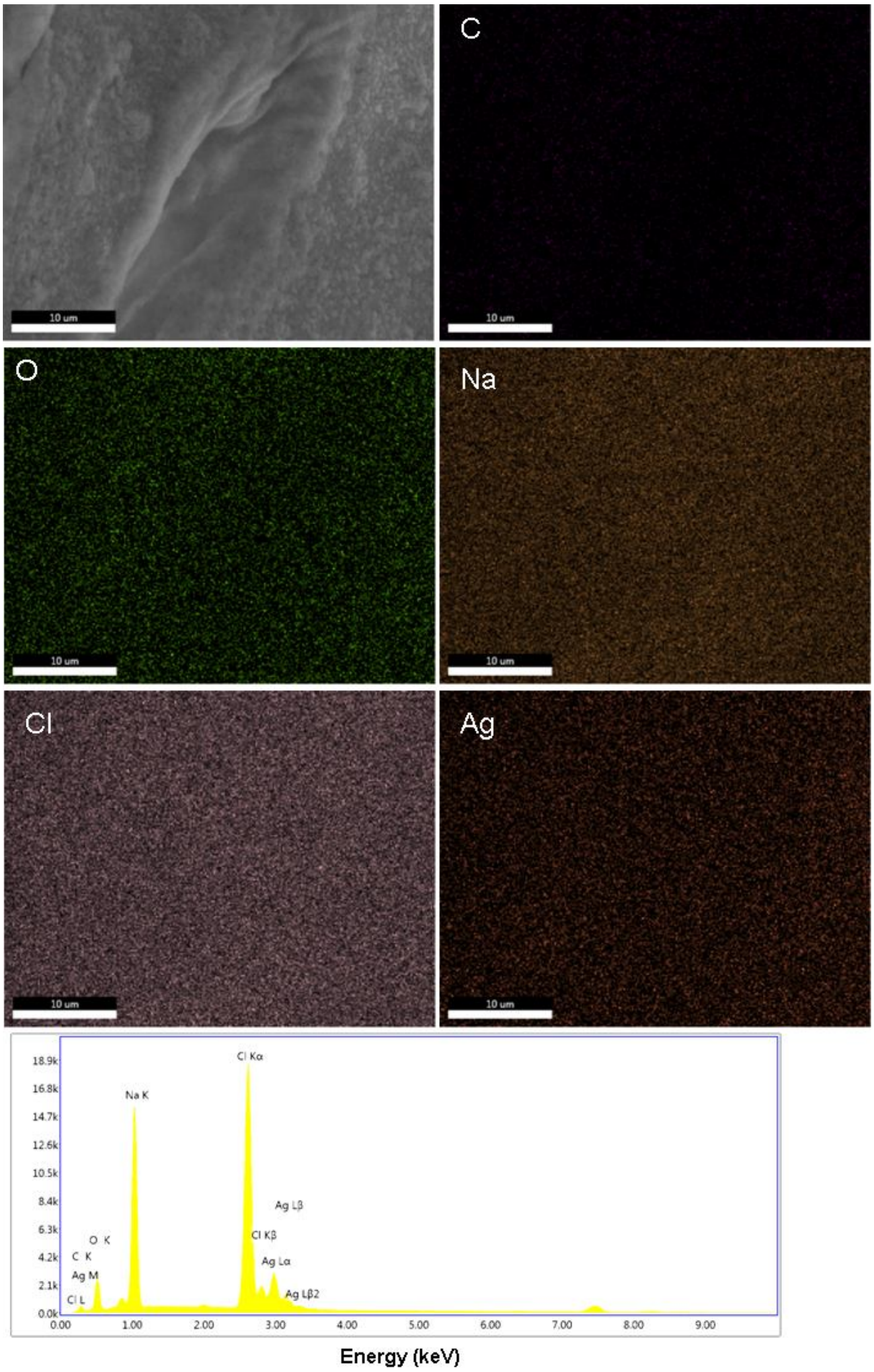

Figure S7. FESEM-EDS compositional elements analysis and mapping images of the Ag-alg capsule exposed to chloride ions.

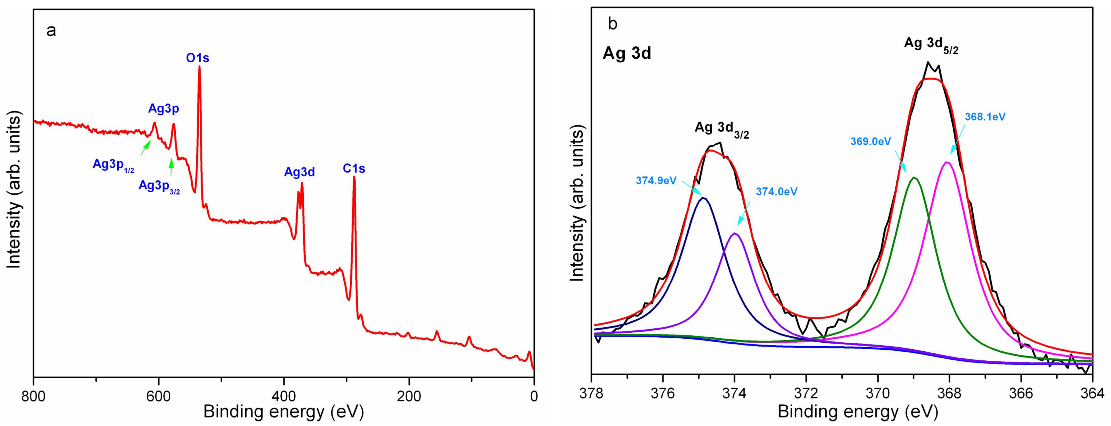

Figure S8. a) XPS survey spectra of Ag-alg. b) High-resolution XPS spectra of Ag 3d in Ag-alg.

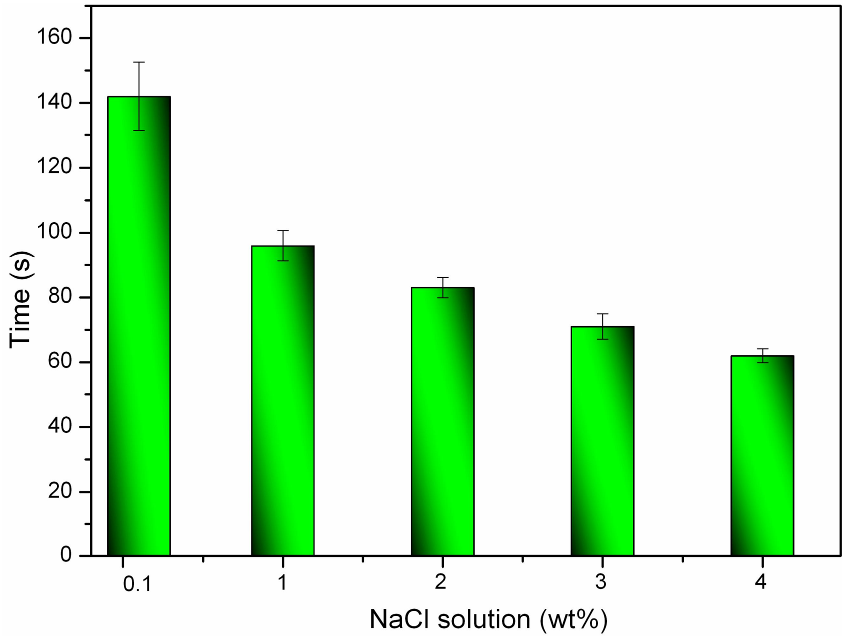

Figure S9. The triggering time of Ag-alginate capsules added with different concentration of NaCl solution.

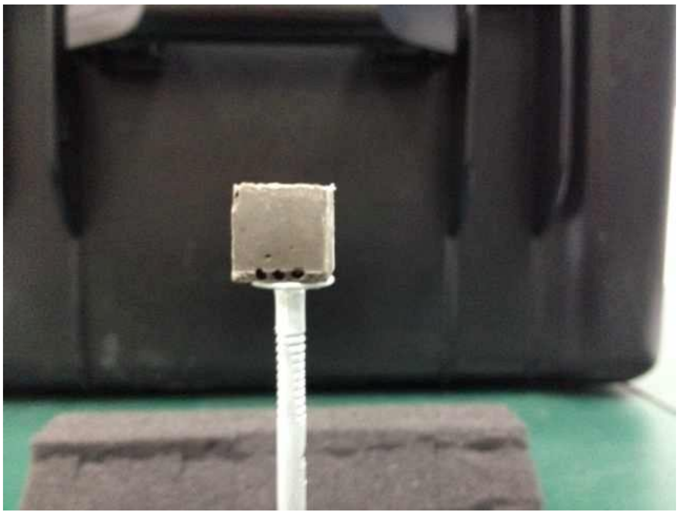

Figure S10. The concrete specimen embedded with chloride ions responsive Ag-alg capsules
